# Erythropoietin‐induced pure red cell aplasia

**DOI:** 10.1002/jha2.481

**Published:** 2022-05-26

**Authors:** Vivian K. P. Yeung

**Affiliations:** ^1^ Department of Pathology Princess Margaret Hospital Kowloon Hong Kong

A 63‐year‐old male with chronic kidney disease on hemodialysis was started on epoetin beta **(Mircera)** for worsening anemia. He achieved good initial response (hemoglobin rose from 6.7 to 12.5 g/dl over 4 months) but developed anemia again with nadir hemoglobin 4.2 g/dl. This was unresponsive to increasing dose of epoetin beta **(Mircera)** or switching to darbepoetin alfa **(Nesp)**.

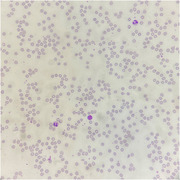

He had anemia (hemoglobin 6.1 g/dl) and reticulocytopenia (reticulocyte 1.3 × 10^9^/L). Iron study was compatible with anemia of chronic illness with hyperferritinemia. Peripheral blood film showed absent polychromasia. Bone marrow examination showed normocellular marrow and absent erythroblasts. Myeloid and megakaryocyte maturation were normal. Blasts were not increased. Dysplastic features and abnormal cellular infiltrates were not found. Autoimmune markers and serology for parvovirus, hepatitis, and HIV were negative. Chest imaging ruled out thymoma. Anti‐erythropoietin antibody was present in high titre (> 1:260) and he was diagnosed with erythropoietin–induced pure red cell aplasia (PRCA).

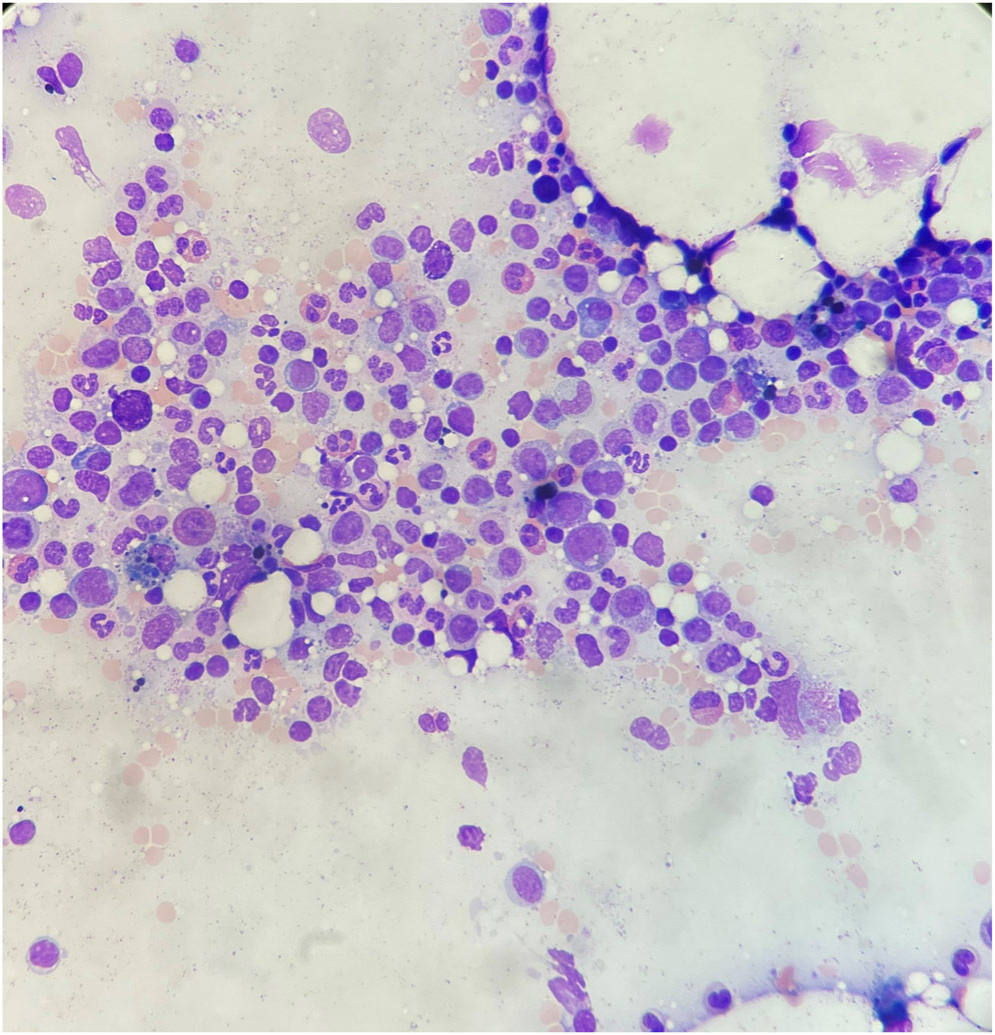

Erythropoietin‐induced antibody‐mediated PRCA is a well‐known but rare complication of erythropoiesis‐stimulating agent (ESA) therapy. The demonstration of absence or near absence of erythroblasts in bone marrow is crucial for its diagnosis. Other characteristics include reticulocytopenia, resistance to ESA therapy, and presence of anti‐erythropoietin neutralizing antibodies. Management includes the cessation of ESA and the use of immunosuppressive treatment.

